# United States Veterinary Medical Association

**Published:** 1892-10

**Authors:** 


### UNITED STATES VETERINARY MEDICAL ASSOCIATION.

The 29th annual meeting of the United States Veterinary Medical Association was convened at Union Hall, Boston, at 10 A. M., Sept. 19th, with the best and most interested attendance of members and visitors that have yet come together. The success of this meeting, which was so eagerly anticipated, was completely fulfilled.

The president, Dr. R. S. Huidekoper, opening his address with a Persian proverb, "A man may be thought clever while he is seeking wisdom; but if he imagines he has found it he is a fool," called attention to the inertia which is apt to follow the enthusiasm of a meeting; reviewed the work which had been accomplished in the last year, and that which had been neglected; and outlined the work in prospect. In regard to the proposed amendment in regard to qualifications for membership, he said:

"You have before you an amendment to the by-laws which establishes a standard of what, it is believed by those who propose it, should be the necessary qualification of education of any Veterinarian who in the future is to become one of us.

"This amendment must not be passed unless we intend that it shall be lived up to, to the very letter of the law.

"If it is passed, it establishes our decision that a course of three years, of at least six months each, is requisite for the proper elementary training of the Veterinarian of the future. If we believe this honestly, then we must vote upon the amendment with equal honesty, and not allow any sympathy or consideration for the effect it may have on any one existing school, it sfuture, finances or any personal matter, to influence our vote.

"Long argument and study of this subject has proven that no governmental legislation or other means can be employed to give us the enforced longer education. We, having founded in this country a representative organization of Veterinarians which is so solidly based that it must remain a governing body, which is attracting the best of the younger men throughout the land, and as seen by our list of applications for membership (which is the public voice of the profession), we are the ones who must determine the standard of veterinary education, which our pioneer work has shown us is demanded for the good of our sons, our successors, and the future. This amendment is an important matter; its passage means that we believe that we must insist that our profession shall have its proper rank and recognition among the other learned ones. The aye or nay of each vote will indicate each individual's estimation of the present standing of his profession."

He called attention to:

"The practical side of veterinary medicine and our relations to the public which have been too much neglected in our meetings. It is just as well that, that other community, the public, from whom we have to earn our own living, should know more of our importance and value, for when it does, it will be profitable to them and we shall earn more from them. Every advance which we make in the estimation of our employer [by showing an increase of scientific knowledge and practical attainment, and every proof which we can furnish of the value of our services, entitle us to charge more for them, and few of us charge enough. We, by our study, our training, and the reputable position which we take as members of this association, thereby acknowledging that our methods of doing business are those ordained by the respectable representatives of the profession, are the natural experts and recognized guardians of the care of animals, in health, sickness and when slaughtered for domestic use in the shape of food and other products. We have a field of labor which can be better appreciated by a few statistics.

"According to the last report of the Department of Agriculture (January and February, 1892), there are now in the United States about fifteen and a half million (15,498,140) horses on farms and ranches, and some two and a third million (2,314,699) mules. This is exclusive of horses in cities; including the latter we can estimate in round numbers about 19,000,000 horses and mules, valued at over one billion of dollars. Sixteen and a half million (16,416,351) milch cows, and over thirty-seven and a half million (37,651,239) oxen and other cattle, make a total of over fifty-four (54,067,590) million head of horned cattle, valued at over nine hundred million dollars. About forty five million (44,938,365) sheep and fifty-two million (52,398,019) swine, together valued at three hundred and fifty million dollars.

"A comparison of the census for the decade from 1880 to 1890, and we can assume that the present increase is as great, shows that the number of horses augmented nearly 50 per cent.; the annual increase for other animals is now estimated at about 2 per cent. for horned cattle; 3 per cent. for sheep, and 3 to 4 per cent. for swine."

He concluded as follows :

"When at Washington last year you honored me with the Presidency of this Association for the third time, I sincerely appreciated your courtesy, but knew that I was receiving more than my share of the reward for the work which so many had done, and I am glad to vacate both the honors and duties, and the latter are not light, to my successor, and promise my cordial aid to the extent of my ability, to both our new officers and to our fraternity in advancing our progress."

The amendment of the constitution (see page 489, September number of JOURNAL), was passed unanimously, with a further amendment that its provisions shall not apply to students who matriculate at Veterinary colleges prior to January, 1893.

The following resolutions were passed :

*Resolved*, That the United States Veterinary Medical Association annually appropriate not less than $150, to be awarded in gold medals, as first and second prizes to the graduates of the year from any recognized veterinary school in the United States, passing the best and second best practical examination. And that a committee of three be appointed by the chair to conduct said examination, the disbursements of said committee to be paid by the society.

L. McLEAN, M.R.C.V.S.
J. F. WINCHESTER.

*Resolved*, That the members of the United States Veterinary

Medical Association here assembled at the annual meeting, desire to express their appreciation and confidence in the work and efforts of the Bureau of Animal Industry, for the advancement of Veterinary Science, the promotion of the agricultural interests of the people, and the elevation of the Veterinary profession; and that the thanks of this association be extended to the chief of that Bureau and his assistants for their efforts.

MADISON BUNKER.

IN MEMORIAM.

WHEREAS: Almighty Providence has seen fit to take trom among us by death, Robert Wood, V. S., and whereas he has as one of the oldest members and organizers of this association ever proven himself an estimable gentleman, an earnest, concientious worker for the elevation of, and a high honor to, our profession.

*Resolved*, That we, members of the U. S. V. M. A., recognize in his death a keen sense of loss to our profession and to this association. His death touches us still more deeply as a personal friend whose loss we shall ever deplore, and whose example in life we shall strive to emulate; and

*Resolved*, That a copy of these resolutions be spread upon the records of our association, and that copies of the same be furnished to the Journal of Comparative Medicine and the American Veterinary Review for publication.

Similar resolutions were passed upon the deaths of the following members: George Bridges, D.V.S.; V. T. Atkinson, V.S., and James L. Kidd, D.V.S.

The election of officers resulted in the choice of :

President, W. L. Williams, V.S., Lafayette, Ind.; Vice President, A. W. Clement, V.S., Baltimore, Md.; Treasurer, James L. Robertson, D.V.S., New York; Secretary, W. Horace Hoskins, D.V.S., Philadelphia.

One hundred and one new members were elected.

The following were placed upon the honorary list of members: A. Chauveau, Director General of the Veterinary Schools of France, proposed by R. S. Huidekoper; William H. Welch, M. D., Professor of Pathology, Johns Hopkins University, proposed by A. W. Clement; and Isiah Michener, the revered and distinguished oldest practioner of Pennsylvania, proposed by W. H. Hoskins.

A resolution was passed to suspend the by-laws in regard to honorary membership for 1893, and appoint a committee of three to nominate for honorary membership three distinguished veterarians from each foreign country, the names to be submitted for election at the Chicago meeting.

It was resolved to term the next meeting "The Thirteenth Annual Meeting of the United States Veterinary Medical Association, and the First International Veterinary Congress of America."

It was gratifying to see old members who have not attended meetings for some years, report and take interest in the meeting, and to find others, who have held aloof, acknowledge their errors, and submit their names for membership. It is to be regretted that the profession from the West was not better represented, but with the election of a western President, it is to be hoped that that section of country will retrieve itself next year. Another notable absence is noticed in regard to the veterinary schools. They of all others should be interested in an association which does so much for the advance of the profession, and yet only one school was properly represented, and that by Liautard and Robertson, who are foundation stones of the association. The school whose graduates furnished the President and Vice-President, and the other large Canadian school, had no representative ; and only one or two of the American schools had a single member of their faculties present.

Tuesday, September 19th, was fully occupied with the reports of the *Comitia Minora* and other committees, and the discussion of these reports and of those. On Wednesday, the morning was occupied with the election of officers. In the afternoon the Massachusetts Association gave a delightful reception and lunch on a steamer which took the party along the coast as far as Beverly. In the evening the association was again the guest of the local members at a theatre party. Thursday was more than occupied with the valuable papers, the subjects of which have already been announced, so that all regretted that another day could not have been added for their discussion. A banquet at Young's Hotel, which was well attended, terminated the meeting. Certain critics regretted that some of the time devoted to entertainment could not have been given to the papers before the association and to longer business meetings, but we believe that the social intercourse and cementing of acquaintanceship does quite as much for the improvement of the profession as the technical work which can be studied over in the library.

